# Type‐specific quantification of particulate methane monooxygenase gene of methane‐oxidizing bacteria at the oxic–anoxic interface of a surface paddy soil by digital PCR

**DOI:** 10.1111/1758-2229.13155

**Published:** 2023-04-20

**Authors:** Rina Shinjo, Fumika Oe, Koki Nakagawa, Jun Murase, Susumu Asakawa, Takeshi Watanabe

**Affiliations:** ^1^ Graduate School of Bioagricultural Sciences Nagoya University, Chikusa Nagoya 464‐8601 Japan; ^2^ School of Agricultural Sciences Nagoya University, Chikusa Nagoya 464‐8601 Japan

## Abstract

Aerobic methane‐oxidizing bacteria (MOB) play an important role in mitigating methane emissions from paddy fields. In this study, we developed a differential quantification method for the copy number of *pmoA* genes of type Ia, Ib, and IIa MOB in paddy field soil using chip‐based digital PCR. Three probes specific to the *pmoA* of type Ia, Ib, and IIa MOB worked well in digital PCR quantification when genomic DNA of MOB isolates and PCR‐amplified DNA fragments of *pmoA* were examined as templates. When *pmoA* genes in the surface soil layer of a flooded paddy were quantified by digital PCR, the copy numbers of type Ia, Ib, and IIa MOB were 10^5^–10^6^, 10^5^–10^6^, and 10^7^ copies g^−1^ dry soil, respectively, with the highest values in the top 0–2‐mm soil layer. Especially, the copy numbers of type Ia and Ib MOB increased by 240% and 380% at the top layer after soil flooding, suggesting that the soil circumstances at the oxic–anoxic interfaces were more preferential for growth of type I MOB than type II MOB. Thus, type I MOB likely play an important role in the methane consumption at the surface paddy soil.

## INTRODUCTION

Methane is one of the potent greenhouse gases, and anoxic environments like natural wetlands, freshwater (lakes and rivers), the gut of termites and ruminant animals, manure, landfills, and paddy fields, are the major sources of atmospheric methane (Canadell et al., 2021). Atmospheric concentrations of methane have increased since the Industrial Revolution. Although the rate of increase of atmospheric methane concentration was temporally paused from the late 1990s to 2006, the rate has increased again since 2007, reaching 1866.3 ppb in 2019 (Canadell et al., 2021). The methane emitted from those anoxic environments is produced in the terminal process of anaerobic decomposition of organic matter by methanogenic archaea, while a considerable amount of produced methane is consumed before it is emitted into the atmosphere by aerobic methane‐oxidizing bacteria (MOB) (Chowdhury & Dick, 2013; Guerrero‐Cruz et al., 2021; Tate, 2015). In paddy fields, 61%–97% and 0%–94% of the methane were estimated to be oxidized in the surface oxic soil layer (Conrad & Rothfuss, 1991) and rice rhizosphere (Denier van der Gon & Neue, 1996; Frenzel et al., 1992; Gilbert & Frenzel, 1995; Goot et al., 2003) before being emitted into the atmosphere, respectively.

Methane‐oxidizing bacteria are the microorganisms that utilize methane as their sole carbon and energy sources, and phylogenetically diverse members of aerobic MOB have been found in *Gammaproteobacteria*, *Alphaproteobacteria*, and *Verrucomicrobia*. These members are often referred to as type I, type II, and, in some cases, type III MOB, respectively, based on the phylogeny, although type I and II MOB have been originally defined based on the physiological, morphological, and chemotaxonomic characteristics, some of which have still been important indicators for typical type I and II MOB (Knief, 2015). According to the grouping by Knief (2015), the type I MOB group consists of diverse methanotrophic members in *Gammaproteobacteria*, which has been further divided into type Ia and Ib clusters in *Methylococcaceae* and type Ic cluster in *Methylothermaceae*. The type II MOB group consists of the methanotrophic members in *Methylocystaceae* and *Beijerinckiaceae*, which are referred to as type IIa and IIb MOB, respectively. The type III MOB are an acidophilic methanotrophic group in *Methyloacidiphilaceae* (Schmitz et al., 2021). In paddy fields, several MOB, which belong to type Ia, Ib, and IIa, have been isolated (Asakawa, 2021).

To reveal the community structure of diverse MOB in the environment, many studies have been reported using molecular biological techniques (Chowdhury & Dick, 2013; Dedysh, 2009; Knief, 2015). Methane monooxygenase (MMO) is the enzyme that catalyses the first step of methane oxidation to methanol in MOB (Murrell et al., 2000). Methane‐oxidizing bacteria possess the membrane‐bound particulate MMO (pMMO), while the soluble MMO (sMMO) is found in type II MOB and some members of the type I MOB (Murrell et al., 2000). One exception is that the genera *Methylocella* and *Methyloferula* in type IIb MOB do not contain pMMO but rather sMMO (Dedysh, 2009; Dedysh et al., 2000; Vorobev et al., 2011). The *pmoA* gene, which encodes the β subunit of pMMO, has been utilized as a suitable functional gene marker to analyse the MOB community since the phylogeny based on the *pmoA* gene roughly corresponds to the 16S rRNA gene‐based phylogeny (Holmes et al., 1995; Kolb et al., 2003). Several universal and group‐specific primers have been designed (McDonald et al., 2008), of which the pair of A189f and A682r (Holmes et al., 1995) and A189f and mb661r (Costello & Lidstrom, 1999) primer sets are the most popular for studies on the diversity and dynamics of the MOB community in environments. In the paddy field ecosystems, the *pmoA* composition of the MOB community has been identified using clone library, terminal restriction fragment length polymorphism (T‐RFLP), and denaturing gradient gel electrophoresis (DGGE) techniques extensively (e.g. reviewed in the study by Conrad, 2007). Next‐generation sequencing of *pmoA* genes (Lüke & Frenzel, 2011) and the total and type‐specific quantification of the copy number of *pmoA* genes using real‐time PCR (Kolb et al., 2003) have also been reported. These studies provided considerable insights into the diversity and dynamics of the MOB community in the paddy field ecosystem. However, further studies are still needed to understand their functions and roles in the ecosystem in more detail, which would provide helpful knowledge for the development of the strategy for the mitigation of methane emission.

One of the preferred habitats of MOB in paddy field is the surface soil layer, where oxic–anoxic interfaces are formed within several millimetres to centimetres in depth (Frenzel et al., 1992). The MOB community (Krause et al., 2010, 2012; Reim et al., 2012) and methane‐driven food web (Murase & Frenzel, 2007) in a soil microcosm, which mimicked the counter‐gradients of O_2_ and methane in the surface soil layer, have been previously analysed by T‐RFLP, microarray, or stable isotope probing‐based techniques. However, these previous studies were conducted by the use of soil obtained from the same paddy field, and the abundance of MOB in the surface layer of flooded paddy soil has not been well studied; only Reim et al. (2012) quantified the copy number of *Methylobacter*‐related *pmoA* genes by competitive T‐RFLP method.

Digital PCR is a burgeoning technique used to quantify the exact number of target molecules. The principle of digital PCR is based on the Poisson statistics; that is, the number of target molecules is estimated from the probability of the presence or absence of amplified fragments from the target molecules in tens of thousands of nanoscale compartments (Quan et al., 2018). The amplified fragments are detected with DNA intercalating dyes (e.g. SYBR green) or fluorescence‐modified probes, which are hydrolyzed by the 5′‐nuclease activity of DNA polymerase during PCR amplification, resulting in the release of the fluorescent molecule. Therefore, the direct counting of amplified fragments and reliable quantification of a low number of target molecules are possible. On the other hand, conventional real‐time PCR is an indirect measurement calibrated with reference standards, which has potential limitations with data accuracy and reproducibility depending on the references used.

The digital PCR technique has been now applied in the studies of environmental microbiology related to biogeochemistry (Shah & Wang, 2019; Voegel et al., 2021). The development of digital PCR quantification of *pmoA* genes must become a useful and powerful technique and provide valuable insights into their ecological significance in the habitats. In the present study, we successfully developed a digital PCR method for quantifying the *pmoA* gene copy number of type Ia, Ib, and IIa MOB in paddy field soil using three type‐specific probes. Optimized conditions were applied in the quantification of the copy number of *pmoA* genes in the thin surface layer of flooded paddy soil, where oxic–anoxic interfaces were formed within the 3‐mm depth. We showed that type I MOB more actively proliferated in the oxic layer than type II MOB.

## EXPERIMENTAL PROCEDURES

### Probe design for the detection of pmoA genes of type Ia, Ib, and IIa MOB

The nucleotide sequences of *pmoA* genes of MOB listed in Knief (2015) and obtained from a paddy soil (Jia et al., 2007) were retrieved from the sequence database at the beginning. The sequences of the target region using the primers A189f and A682r (Holmes et al., 1995) were aligned with ClustalW on MEGA10.1.7 for mac (Stecher et al., 2020), followed by a manual searching of candidate regions for the design of probes, which are specific to type Ia, Ib, and IIa MOB. The specificity of the candidate nucleotide sequences was further checked using the *pmoA* gene sequences and related gene sequences (e.g. *amoA*) of isolates, retrieved from the FunGene website (Fish et al., 2013). In total, 606 sequences were used for probe design. Three probes, mb661_Ia, Ib547, and II646_mod, targeting the *pmoA* genes of type Ia, Ib, and IIa MOB, respectively, were used in the present study (Table 1). All probes were designed to hybridize to the sense strand of the target DNA.

**TABLE 1:** The probe features used in this study.

Probe	Target	Sequence (5′–3′)	Length (mer)	*T*m (°C)	Annealing temp (°C)	Mismatch in targets				Total
						0	1	2	>3	
mb661_Ia	Ia	ATACWGGAGCAACGTCYTTACCRA	24	59	57	24	13	1	1	39
Ib547	Ib	ACATCAGCATSCCGTTGTAYTCMAC	25	64	55	29	28	7	1	65
II646_mod	IIa	CGTGCCGCGCTCSACCATGY	20	70	57	197	3	1	1	202

*Note*: *T*m values were calculated, based on the Primer Express Software (Thermo Fisher Scientific).

The designed probes were synthesized by the Integrated DNA Technologies (Coralville, USA), for which a modification was made with a fluorophore of 6‐FAM™ (6‐Carboxyfluorescein; absorbance max, 495 nm; emission max 520 nm) or Yakima Yellow® (absorbance max, 525 nm; emission max 549 nm) on the 5′‐terminal nucleotide and a double quencher of ZEN™ and Iowa Black® FQ (IBFQ) on the internal and 3′‐terminal nucleotides.

### Template DNAs used for the digital PCR quantification

The genomic DNA of *M. koyamae* Fw12E‐Y^T^ (type Ia; Ogiso et al., 2012), *M. ishizawai* RS11D‐Pr^T^ (type Ib; Khalifa et al., 2015), *Methylogaea oryzae* E10^T^ (type Ib; Geymonat et al., 2011), *Methylocystis echinoides* IMET10491^T^ (type IIa; Bowman et al., 1993; Gal'chenko et al., 1978), *M. bryophila* H2s^T^ (type IIa; Belova et al., 2013), and *Methylocystis* sp. SS37A‐Re isolated from a Japanese paddy field (type IIa; Dianou et al., 2012) were examined for digital PCR quantification.

PCR amplicons obtained from *Methylosinus trichosporium* OB3b^T^ (type IIa; Bowman et al., 1993; Whittenbury et al., 1970) and *Methylobacter luteus* NCIMB 11914^T^ (type Ia; Bowman et al., 1993) with the primer set A189f and A682r, PCR amplicons of *pmoA2* gene derived from *M. bryophila* H2s^T^ and *Methylocystis* sp. SS37A‐Re, and *pmoA*‐, *pxmA*‐, and *amoA*‐like clones obtained from a clone library derived from a Japanese paddy field soil were also used for the examination. Primers designed for the flanking region of *pmoA2* in *M. bryophila* H2s^T^ and *Methylocystis* sp. SS37A‐Re were used to amplify the *pmoA2* gene in the present study. The PCR products were purified by using the NucleoSpin® Gel and PCR Clean‐up kit (Machery‐Nagel, Düren, Germany) before use as a template DNA for digital PCR. A clone library was constructed from the PCR products amplified from Japanese paddy soil DNA (Liu et al., 2018) using A189f and A682r. The ligated pT7 Blue T‐vectors were transformed into the competent cells of *Escherichia coli* DH5α. Transformed cells were grown in the Luria‐Bertani medium, and plasmid DNAs containing a DNA fragment of *pmoA* were extracted by using NucleoSpin® Plasmid EasyPure (Machery‐Nagel). Sequencing analysis was outsourced to the DNA sequencing service of Eurofins Genomics Japan (Tokyo, Japan). The accession numbers of sequences determined in the present study were LC716069–LC716108 and LC744973.

The copy number of the target genes in the genomic DNA, PCR amplicons, and clones (plasmid DNA) was calculated from the DNA concentrations with their size (length) information. The concentrations were quantified with a Qubit Fluorometer (Thermo Fisher, Waltham, USA).

### Paddy soil samples for the digital PCR quantification

To quantify the copy number of *pmoA* genes in paddy field soil by digital PCR, DNA extracted from the surface soil layer of flooded paddy soil (Nakagawa et al., 2020) was used for the examination. The details of the experiment and sampling of each layer have been previously described (Nakagawa et al., 2020). In brief, paddy soil, obtained from a paddy field at the Aichi Agricultural Research Center, Japan, was statically incubated in triplicate microcosms under atmospheric, dark and flooded conditions for 30 days. That is, the surface of flooded water had contacted with atmospheric air during the incubation. The soil was mixed with 0.2% (w/w) of pulverized rice straw before incubation. After 30 days under the flooding conditions, the O_2_ concentration and the amount of ferrous iron (Fe(II)) were quantified; a steep decrease in dissolved O_2_ concentrations was observed within the 3 mm depth, while the amount of Fe(II) was the lowest in the top layer and increased with the depth. Five soil layers (0–2, 2–4, 4–6, 6–8, and 8–10 mm in depth) were obtained by slicing the soil profile with a knife, DNA was then extracted using ISOIL for Beads Beating (Nippon Gene, Tokyo, Japan). The bulk soil before incubation (flooding) was also used for the analysis.

### Digital PCR quantification of the pmoA gene

The QuantStudio™ 3D Digital PCR System (Thermo Fisher Scientific) was used to quantify the copy number of *pmoA* genes. The 15 μL of reaction mixture consisted of 7.5 μL of 2 × QuantStudio™ 3D Digital PCR Master Mix v2, each 1.35 μL of each primer, A189f and A682r (each 10 μM), 0.75 μL of the probe (5 μM), 1.5 μL of template DNA, 1.0 μL of bovine serum albumin (BSA, 20 mg mL^−1^; TaKaRa, Kusatsu, Japan), and 1.55 μL of sterilized ultrapure water, and 14.5 μL of the mixture was loaded onto a QuantStudio™ 3D Digital PCR 20 K Chip v2 using the Chip Loader. The chip was made from a silicon substrate and contained 20,000 nanoscale reaction wells. When the copy number of *pmoA* genes was quantified with the probes Ib547 and II646_mod, dimethyl sulfoxide (DMSO) was also added to the reaction mixture at a final concentration of 2.0% because the fluorescence intensity of positive wells (targets) was enhanced (data not shown). After loading the reaction mixture, the surface of the wells was immediately covered with an immersion fluid and sealed with a lid cover. The prepared chips were set in the ProFlex™ PCR system (Thermo Fisher Scientific) for the PCR amplification.

The PCR program consisted of initial denaturation at 96.0°C for 10 min and 39 cycles of annealing and elongation at 55–57°C for 3 min, and denaturation at 96.0°C for 30 s, followed by the final annealing and elongation at 55–57°C for 3 min and then a cooling phase to 10°C. The annealing and elongation temperatures varied depending on the probes used (Table 1). The fluorescence intensity of each well after PCR was measured using the QuantStudio™ 3D Digital PCR System. The data were analysed using the QuantStudio™ 3D AnalysisSuite™ Cloud Software. The criteria for the positive or negative wells were based on the fluorescence intensity of the wells. In the present study, the default setting (automatic counting) was applied to the counting of the number of positive wells, except for some minor cases when the counting by the default setting was not reflected by the distribution of fluorescence intensity. In that case, the criteria were determined manually by checking the distribution. The final copy number of the targeted *pmoA* genes in a template was estimated using the Poisson Plus model (Majumdar et al., 2017) from the counted number of positive wells.

### Real‐time PCR quantification of the pmoA gene

The total copy number of *pmoA* genes in the paddy soil samples was quantified using the SYBR‐Green method, as described previously (Kolb et al., 2003; Liu et al., 2016). Serial dilutions of the genomic DNA from *Methylocystis* sp. SS37A‐Re were used as the reference standard, where the copy number of the *pmoA* gene was estimated from the concentration and size of genomic DNA. The 25 μL of reaction mixture consisted of 12.5 μL of 2 × TB green® Premix Ex Taq™, each 0.5 μL of each primer A189f and mb661 (each 10 μM), 1.0 μL of BSA (20 mg ml^−1^), 2.0 μL of template DNA, and 8.5 μL of sterilized ultrapure water. The program consisted of initial denaturation at 95°C for 10 s, 45 cycles of denaturation at 95°C for 30 s, annealing at 65.5°C for 30 s, and elongation at 72°C for 45 s.

### Statistical analysis

To evaluate the precision of digital PCR quantification, the coefficient of variation (CV) of the quantified values was calculated as follows:

(1) CV=standard deviation/average

The copy number of the *pmoA* genes among the layers of flooded paddy soil was logarithmically converted, followed by examination with the Tukey–Kramer's test.

## RESULTS AND DISCUSSION

### In silico analysis of designed probes

In the present study, three probes, mb661_Ia, Ib547, and II646_mod, which were designed newly or modified from previously known primers, were used for type‐specific quantification of the copy number of *pmoA* genes (Table 1 and Table S1). The *pmoA* genes of the MOB isolates (61 sequences) and some paddy soil clones obtained in the present study (24 sequences) and a previous study (36 sequences, AB222897–AB222932; Jia et al., 2007) were aligned to design the probes. The specificity of the probe sequences was further checked using the *pmoA* gene sequences and related gene sequences (e.g. *amoA*) of isolates, retrieved from the FunGene website (Fish et al., 2013). In total, 606 sequences were used for the probe design (Table S1). The *pmoA* sequences from many other uncultured groups were not considered in these probe designs since their functions have not been demonstrated in most cases (Knief, 2015) and it was difficult to find the conserved regions.

The type Ia group consists of diverse members of MOB. We examined the primer Mb601R (5′ ACRTAGTGGTAACCTTGYAA 3′) with some modifications, which was originally designed to detect the *Methylobacter/Methylosarcina* groups in type Ia MOB (Kolb et al., 2003), as a candidate probe (Mb601_mod, 5′ ACRTARTGRTAACCTTG 3′) for the digital PCR quantification at the beginning. However, target *pmoA* genes were not detected with the probe Mb601_mod (data not shown), probably because of the lower *T*m value of Mb601_mod (47°C), compared with that of the primer set A189f (53°C) and A682r (51°C). To detect fluorescence derived from target sequences in the cycle of PCR amplification, probe must be hybridized before primer annealing and elongation. That is, the lower *T*m value of Mb601_mod is not suitable for the probe. Therefore, the probe mb661_Ia (*T*m, 59°C) was designed from the region of the *pmoA* gene corresponding to the primer mb661 (5′ CCGGMGCAACGTCYTTACC 3′) (Costello & Lidstrom, 1999), which is often used to study environmental MOB. The sequence of the probe mb661_Ia was determined by the modification of mb661 based on the *pmoA* sequences of *Methylicorpusculum*, *Methylobacer*, *Methyloglobulus*, *Methylomarinum*, *Methylomicrobium*, *Methylomonas*, *Methyloprofundus*, *Methylosarcina*, *Methylosoma*, *Methylotuvimicrobium*, and *Methylovulum* (Table S1). The sequence of mb661_Ia was identical to the targeted region of 24 out of the 39 examined *pmoA* sequences of type Ia MOB and had one mismatched position in 13 out of the 39 examined *pmoA* sequences. More than two mismatched positions were found between the sequences of the probe and most non‐target *pmoA* genes (Table S1).

The probe Ib547 for detecting type Ib MOB was newly designed from the *pmoA* sequences of *Methylocaldum*, *Methylococcus*, *Methylogaea*, *Methylomagnum*, *Methyloparacoccus*, and *Methyloterricola*. Some clones, known as Rice Paddy Cluster 1 (RPC1) or freshwater sediment‐1 (Lüke et al., 2010), were also included in the alignment for this probe design. The sequence of the probe Ib547 was identical to the targeted region of 29 out of the 65 examined sequences of the type Ib group (27/33 sequences other than RPC1). One mismatched position was observed in 28 out of the 66 sequences, many of which were derived from the sequences of the RPC1 cluster (23/33 RPC1 sequences). The sequence of Ib547 was identical to the *pmoA* gene of type Ic MOB in addition to type Ib MOB (Table S1), but the members of type Ic generally inhabit thermal and/or saline environments (Hirayama, 2016), and the related *pmoA* sequences have not been retrieved from paddy field soils (Lüke et al., 2010; Lüke & Frenzel, 2011). Therefore, the risk of the simultaneous detection of type Ib and Ic in paddy field environments was considered to be minimal.

Type IIa MOB consisted of *Methylosinus* and *Methylocystis*. Probe II646_mod was modified from the primer II646R (5′ CGTGCCGCGCTCGACCATGYG 3′), which was previously designed to detect the genera (Kolb et al., 2003). The in silico comparison with the target *pmoA* genes revealed that the sequence of II646_mod was identical to the target region of *pmoA* for most type IIa MOB (197/202 *pmoA* sequences). The probe had one or two mismatches with most of the *pmoA2* sequences of type IIa (14/19 *pmoA2* sequences) and more than one mismatch with the *pmoA* gene sequences derived from type IIb MOB belonging to *Beijerinckiaceae* (Table S1), inhabiting moderately acidic environments (Hakobyan & Liesack, 2020). Since type IIb MOB are a minor group in paddy field environments (Kolb et al., 2003; Lüke et al., 2010; Lüke & Frenzel, 2011), the *pmoA* detected with the probe II646_mod in paddy field environments is assumed to be mainly derived from type IIa MOB.

All probes had many mismatches with the gene sequences in *pxmA* and *amoA* clusters, which is a homologue of *pmoA* found in MOB and another homologue encoding the subunit A of ammonia monooxygenase found in ammonia oxidizers, respectively (Table S1). Therefore, the *pxmA* and *amoA* genes were unlikely to be detected by the examined probes irrespective of the primers used for PCR.

### Optimization of digital PCR conditions

PCR was performed using the primer set A189f and A682r (Holmes et al., 1995), which has been used as a universal primer set for the PCR amplification of *pmoA* and *amoA*. The lower *T*m value of A682r (51°C) was more suitable for the probe hybridization (*T*m of designed probes, 59–70°C) than mb661 (Costello & Lidstrom, 1999), another universal primer with a higher *T*m value (58°C). We selected primer A682r for this reason, although advantages and disadvantages have been reported for both primers in the diversity analysis of *pmoA* (Bourne et al., 2001; Lüke et al., 2010; McDonald et al., 2008). The annealing temperature of the digital PCR was optimized based on the *T*m value of the probes for the primer set A189f and A682r (Table 1). An attempt was made to optimize the number of PCR cycles, but the sensitivity (the number of positive wells) did not improve significantly. When the PCR program was changed from two‐step to three‐step program, the sensitivity was significantly decreased (data not shown).

**FIGURE 1:**
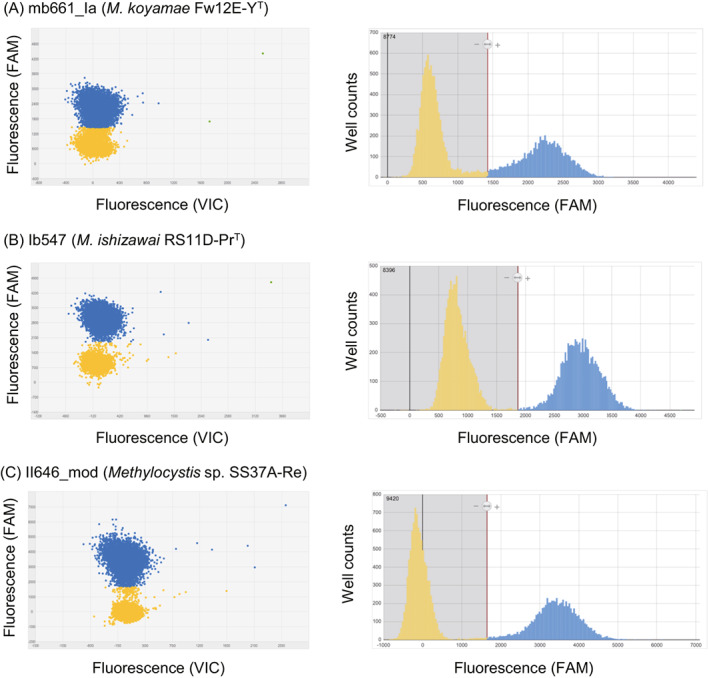
Representative examples of scatterplot (left) and histogram (right) by digital PCR with FAM‐labelled probes (A) mb661_Ia, (B) Ib547, and (C) II646_mod, respectively. The blue and yellow plots indicate the positive and negative wells, respectively.

Figure 1 shows representative examples of the digital PCR results. Under the optimized PCR conditions, the fluorescence intensity of the wells increased when the target *pmoA* genes were amplified and the probe was effectively hybridized with the amplified fragments during PCR, resulting in a clear separation of the fluorescence intensity between the wells in the presence and absence of the target *pmoA* genes. On the other hand, the fluorescence intensity did not increase even when non‐target *pmoA* genes were amplified because hybridization of the probe did not occur (data not shown).

Table 2 shows a summary of the digital PCR quantification. All the probes were effectively hybridized with the amplified fragments when the probe sequence was identical to the target sequences. The ratio of the logarithmic value of the estimated copy number to the logarithmic value of the predicted copy number ranged from 0.79 to 1.09 when all targeted *pmoA* genes were examined. In contrast, positive wells were scarcely detected from non‐target *pmoA* genes, which had three or more mismatches with the probes. Thus, the specificity of the probes was sufficiently high to detect the target *pmoA* genes. Some MOB possess two or three sets of *pmoA* genes in their genome, and the designed probe is not always identical to all the multiple *pmoA* gene sets; one mismatched position exists between the probe II646_mod and *pmoA2* of *Methylocystis* sp. SS37A‐Re (type IIa; Dianou et al., 2012) and *Methylocystis bryophila* H2s^T^ (type IIa; Belova et al., 2013) (Table 2). However, the copy numbers estimated by the digital PCR quantification would not be influenced by such mismatches, since *pmoA* genes other than *pmoA2* gene in the genomic DNA should be amplified in a well. This suggests that the values obtained by digital PCR are equivalent to the exact abundance of MOB, which may be an advantage of digital PCR over real‐time PCR for quantification.

**TABLE 2:** Specificity of digital PCR quantification of *pmoA* genes with type‐specific probes.

Type	Template			*pmoA* (copies)	Primer mismatch (forward/reverse)	Probe mb661_Ia		Probe Ib547		Probe II646_mod	
	Species	DNA type	Accession number			Mismatch	Sensitivity	Mismatch	Sensitivity	Mismatch	Sensitivity
Ia	*Methylomonas koyamae* Fw12E‐Y^T^	genome DNA	GCA_001312005.1	1	0/2	0	+	3	−	7	−
	*Methylobacter luteus* NCIMB 11914^T^	PCR amplicon	LC744973	1	n.d./0	0	+	5	−	7	−
	clone 273		LC716069	1	0/0	0	+	3	−	7	n.e.
Ib	*Methylomagnum ishizawai* RS11D‐Pr^T^	genome DNA	GCA_019670005.1	2	0/1	5	−	0	+	4	−
					0/1	5		0		4	
	*Methylogaea oryzae* E10^T^	genome DNA	GCA_019669985.1	2	0/2	3	n.e.	0	+	6	n.e.
					0/2	3		0		6	
	RPC1 (clone 300)	plasmid	LC716074	1	0/0	3	n.e.	3	−	5	n.e.
IIa	*Methylocystis* sp. SS37A‐Re	genome DNA	GCA_027925385.1	3	0/1	5	−	12	−	0	+
					0/1	5		12		0	
					1/1 (*pmoA2*)	6		10		1	
	*Methylosystis echinoides* IMET10491^T^	genome DNA	GCA_027923385.1	2	0/1	5	n.e.	11	n.e.	0	+
					0/1	5		11		0	
	*Methylocystis bryophila* H2s^T^	genome DNA	GCA_027925445.1	3	0/1	5	n.e.	9	n.e.	0	+
					0/1	5		9		0	
					0/1 (*pmoA2*)	5		8		1	
	*Methylosinus tricosporium* OB3b^T^	PCR amplicon	GCA_002752655.1	1	0/1	5	n.e.	9	n.e.	0	+
					0/1	5		9		0	
IIa (*pmoA2*)	*Methylocystis* sp. SS37A‐Re	PCR amplicon	GCA_027925385.1	1	1/1	6	−	10	−	1	+
	*Methylocystis bryophila* H2s^T^	PCR amplicon	GCA_027925445.1	1	0/1	6	−	8	−	1	+
Other	*pxmA* (clone 248)	Plasmid	LC716097	1	0/0	12	−	11	−	3	−
Other	*amoA* (clone 305)	Plasmid	LC716098	1	0/0	14	−	11	−	7	−

Abbreviations: n.e., not examined; n.d., no data.

### Validity of digital PCR quantification for low copy numbers of pmoA gene

**FIGURE 2:**
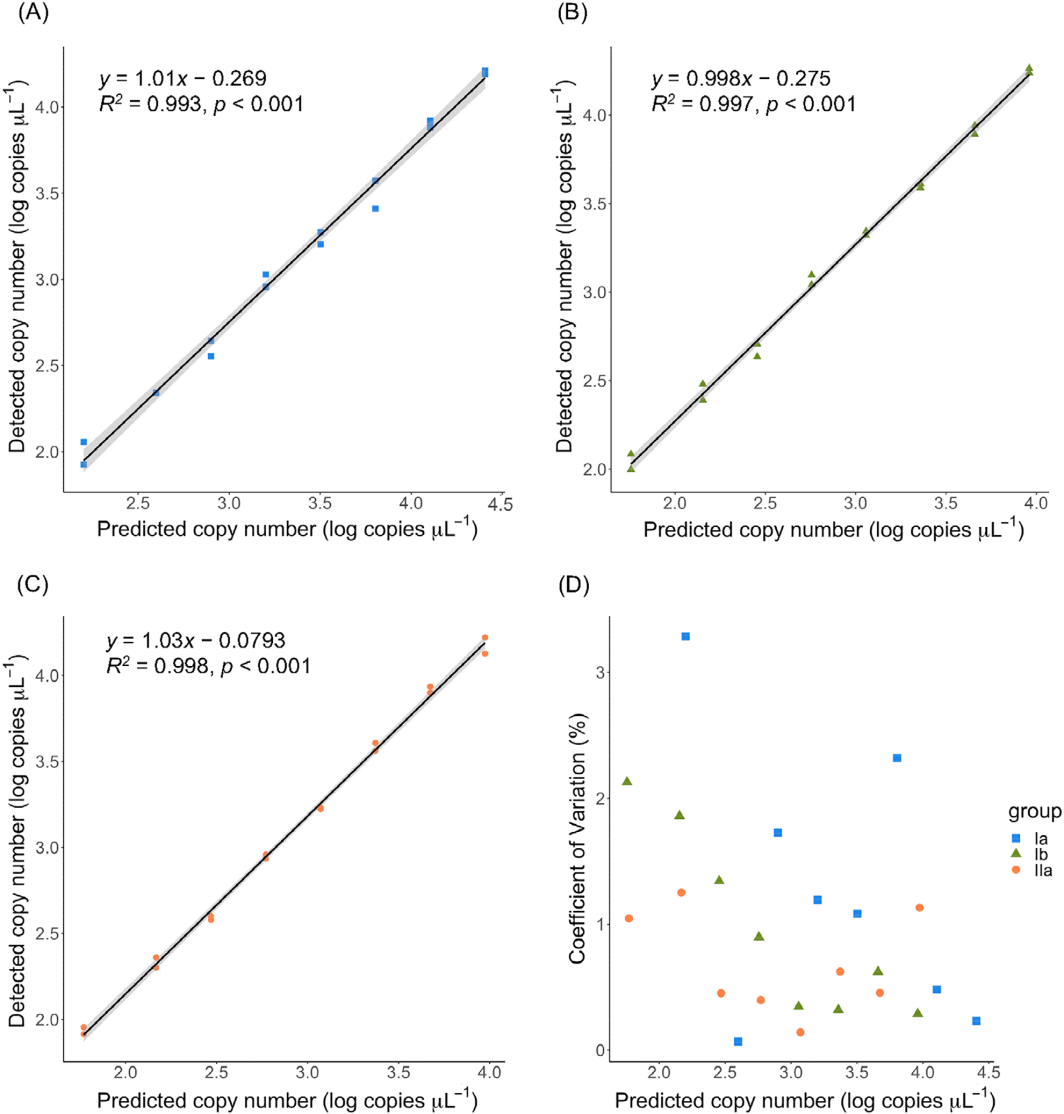
Relationships between the estimated and predicted copy numbers of serially diluted genomic DNA of (A) *M. koyamae* Fw12E‐Y^T^ (type Ia), (B) *M. ishizawai* RS11D‐Pr^T^ (type Ib), (C) *Methylocystis* sp. SS37A‐Re (type IIa), and (D) Coefficient of variation of two quantified values. Grey zones indicate the 95% confidence interval.

The precise quantification of a low number of target molecules is also an advantage of digital PCR quantification. To evaluate precision, serial dilutions of genomic DNA (from ca. 5 × 10^1^ to 10^4^ copies of *pmoA* μL^−1^) of *Methylomonas koyamae* Fw12E‐Y^T^ (type Ia; Ogiso et al., 2012), *Methylomagnum ishizawai* RS11D‐Pr^T^ (type Ib; Khalifa et al., 2015), and *Methylocystis* sp. SS37A‐Re (type IIa; Dianou et al., 2012) were quantified using the probes mb661_Ia, Ib547, and II646_mod, respectively, by digital PCR (Figure 2). A good linear relationship (*R* ^2^ > 0.993) was observed with a narrow 95% confidence interval between the quantified and expected copy numbers in either case. The coefficient of variation was <4% irrespective of the probes and the copy number of templates, indicating that precise quantification is possible even for the samples with a low number of targets. These results suggested that digital PCR quantification of *pmoA* genes is a powerful tool when the abundance of MOB is low in the habitats.

### Quantification of pmoA genes in paddy field soil

The copy number of *pmoA* genes of type Ia, Ib, and IIa MOB in the thin surface soil layer of a flooded paddy was quantified at a depth interval of 2 mm (0–2, 2–4, 4–6, 6–8, and 8–10 mm in depth) using the optimized digital PCR conditions. The samples of each soil layer were obtained from a microcosm experiment, the soil of which was incubated in an open container under dark and flooded conditions for 30 days (see the Experimental procedures). At that time, dissolved O_2_ was depleted within 3‐mm depth, while the contents of Fe (II) increased with depth (Nakagawa et al., 2020). Nakagawa et al. (2020) analysed the vertical distribution of *Gallionella*‐related iron‐oxidizing bacterial communities (*Gallionella*‐related FeOB) in the same soil samples, most of which were microaerophiles, and showed that the copy number of 16S rRNA genes of *Gallionella*‐related FeOB was highest at the topsoil layer. Although methane produced in or released from the soil was not measured in this study, Baba et al. (2016) observed that active methane production was occurred within a few days when the same soil was incubated under anoxic conditions. These previous results indicated that counter‐gradients of O_2_ and methane were formed in the surface layer of incubated soil.

**FIGURE 3:**
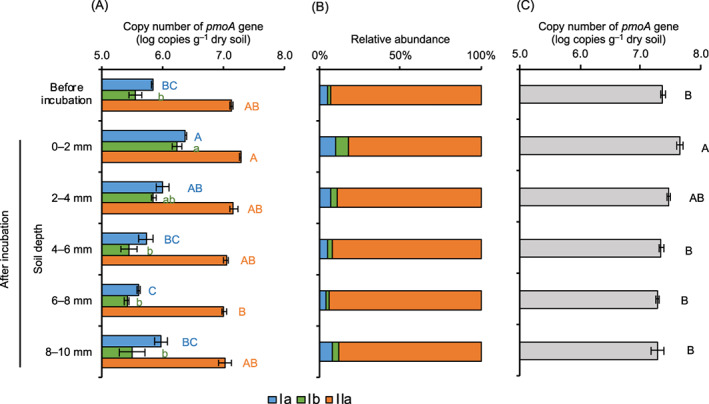
The copy numbers of *pmoA* genes in the thin surface layer of flooded paddy soil (0–2, 2–4, 4–6, 6–8, and 8–10 mm in depth) quantified by digital PCR and real‐time PCR. (A) The copy numbers of *pmoA* genes of type Ia, Ib, IIa MOB. (B) Relative abundance of type Ia, Ib, IIa MOB. (C) The total copy numbers of *pmoA* genes quantified by real‐time PCR. ‘Before incubation’ indicates the bulk soil before flooding. Bars show the standard errors of the mean (*n* = 3). Different coloured letters indicate that the values are significantly different among the samples (*p* < 0.05).

The copy numbers of *pmoA* genes of type Ia, Ib, and IIa MOB in the soil layers were ca. 10^5^–10^6^, 10^5^–10^6^, and 10^7^ copies g^−1^ dry soil, respectively (Figure 3). The number of all types of MOB was the highest in the topsoil layer (0–2 mm in depth) although the number of type IIa MOB at the topsoil layer was not significantly different from those before incubation and in the deeper soil layers except for 6–8 mm depth. Since the soil below 3 mm depth was almost anoxic (Nakagawa et al., 2020), MOB proliferated at the oxic zone using methane, which was produced by methanogenic archaea in the deeper anoxic zone. The copy numbers of type Ia and Ib MOB at the top layer increased by 240% and 380% after the soil was flooded, respectively. These results indicated that type Ia and Ib MOB are likely key players in the consumption of methane at the surface of paddy field soil. Earlier works (Krause et al., 2010, 2012; Reim et al., 2012) also drew a similar conclusion, but this is the first study that showed the type‐specific proliferation of MOB in the thin surface soil quantitatively. Type I MOB likely has some advantages over type II MOB for growth in environments under very low O_2_ concentrations (Knief, 2019). Thus, the soil circumstances at the oxic–anoxic interfaces were more preferential for the growth of type I MOB than type II MOB, as the substrate for MOB (i.e. methane) was continuously supplied from the anoxic zone and was not a limiting factor.

The copy number of total *pmoA* genes quantified using real‐time PCR with the primer set A189f and mb661r (Costello & Lidstrom, 1999; Kolb et al., 2003) was 1.9 × 10^7^–4.6 × 10^7^ copies g^−1^ dry soil with the highest number in the topsoil layer (Figure 3). The sum of the *pmoA* copy numbers of type Ia, Ib, and IIa MOB quantified by the digital PCR (1.1 × 10^7^–2.3 × 10^7^ copies g^−1^ dry soil) was close to the total copy numbers quantified using the real‐time PCR, suggesting that the digital PCR conditions presented in the current study are applicable to the quantification of *pmoA* genes in the paddy field soil.

In conclusion, we successfully proposed digital PCR conditions for the type‐specific quantification of *pmoA* genes of MOB. The designed probes, mb661_Ia, Ib547, and II646_mod, were suitable for quantifying the *pmoA* genes of type Ia, Ib, and IIa MOB in paddy field soil. The absolute quantification of each type of MOB by digital PCR showed the proliferation of type Ia and Ib MOB at the surface oxic layer of paddy soil developed after flooding. The absolute quantification of MOB by digital PCR would facilitate comparisons between different researches, which leads to the update of our understanding of the ecology of MOB in the paddy field ecosystem. The optimized PCR conditions can be applicable to the ecological studies of MOB in other environments if the *pmoA* of targets is covered by the detection range of the probes. As several probes have been designed for the microarray analysis (Bodrossy et al., 2003; Stralis‐Pavese et al., 2004), the development of genus‐ or species‐specific probes will enable to reveal more detailed dynamics of MOB in environments in the future. Hence, the digital PCR can be a useful and powerful method to understand the ecology of MOB communities in the environment.

## AUTHOR CONTRIBUTIONS

**Rina Shinjo:** Formal analysis (equal); investigation (equal); writing – original draft (equal); writing – review and editing (equal). **Fumika Oe:** Investigation (equal); writing – review and editing (equal). **Koki Nakagawa:** Investigation (supporting); resources (equal). **Jun Murase:** Resources (equal); writing – review and editing (equal). **Susumu Asakawa:** Funding acquisition (equal); writing – review and editing (equal). **Takeshi Watanabe:** Methodology (equal); writing – original draft (equal); writing – review and editing (equal).

## CONFLICT OF INTEREST STATEMENT

The authors declare no conflicts of interest associated with this manuscript.

## Supporting information


**Table S1.** The specificity of designed probes to the targeted *pmoA* genes. Nucleotides that are identical and mismatched to the probe are indicated in yellow and blue, respectively. Sequences belonging to the taxonomic group targeted by each probe are marked with red squares.

## Data Availability

The GenBank/EMBL/DDBJ accession numbers for the nucleotide sequences determined in this study are LC716069–LC716108, and LC744973.
